# Rho GTPases in Intellectual Disability: From Genetics to Therapeutic Opportunities

**DOI:** 10.3390/ijms19061821

**Published:** 2018-06-20

**Authors:** Valentina Zamboni, Rebecca Jones, Alessandro Umbach, Alessandra Ammoni, Maria Passafaro, Emilio Hirsch, Giorgio R. Merlo

**Affiliations:** 1 Department Molecular Biotechnology and Health Science, University of Torino, Via Nizza 52, 10126 Turin, Italy; valentina.zamboni@unito.it (V.Z.); rebecca.jones@edu.unito.it (R.J.); alessandro.umbach@edu.unito.it (A.U.); alessandra.ammoni@edu.unito.it (A.A.); emilio.hirsch@unito.it (E.H.); 2 National Research Council (CNR) Institute for Neuroscience, Via Luigi Vanvitelli, 32, I-20129 Milan, Italy; m.passafaro@in.cnr.it

**Keywords:** RhoA, Rac1, cdc42, intellectual disability, neuronal networks, GTPase pathway

## Abstract

Rho-class small GTPases are implicated in basic cellular processes at nearly all brain developmental steps, from neurogenesis and migration to axon guidance and synaptic plasticity. GTPases are key signal transducing enzymes that link extracellular cues to the neuronal responses required for the construction of neuronal networks, as well as for synaptic function and plasticity. Rho GTPases are highly regulated by a complex set of activating (GEFs) and inactivating (GAPs) partners, via protein:protein interactions (PPI). Misregulated RhoA, Rac1/Rac3 and cdc42 activity has been linked with intellectual disability (ID) and other neurodevelopmental conditions that comprise ID. All genetic evidences indicate that in these disorders the RhoA pathway is hyperactive while the Rac1 and cdc42 pathways are consistently hypoactive. Adopting cultured neurons for in vitro testing and specific animal models of ID for in vivo examination, the endophenotypes associated with these conditions are emerging and include altered neuronal networking, unbalanced excitation/inhibition and altered synaptic activity and plasticity. As we approach a clearer definition of these phenotype(s) and the role of hyper- and hypo-active GTPases in the construction of neuronal networks, there is an increasing possibility that selective inhibitors and activators might be designed via PPI, or identified by screening, that counteract the misregulation of small GTPases and result in alleviation of the cognitive condition. Here we review all knowledge in support of this possibility.

## 1. Introduction

Small GTPases of the Rho class comprise a set of highly conserved signaling GTPases, including RhoA, RhoB and RhoC (the Rho subclass), Rac1, Rac2, Rac3 and RhoG (the Rac subclass) and cdc42, TC10/RhoQ and TCL/RhoJ (the cdc42 subclass). The most extensively studied members of the Rho family in the nervous system are RhoA (ras homologous member A), Rac1 (ras related C3 botulinum toxin substrate 1) and cdc42 (cell division cycle 42) [1].

Similar to other signaling GTPases, they cycle between GTP (active) and GDP (inactive)-bound states. The GTP/GDP cycle is regulated by complex protein:protein interactions (PPI) between the GTPase and various partners that either increase (Guanine nucleotide Exchange Factors, GEFs) or decrease (GTPase-activating proteins, GAP) their function to activate downstream targets.

Although the GTPase has an intrinsic enzymatic ability to cleave GTP to GDP, switching in the inactive state, commonly we refer to hyper- and hypo-active GTPase as its increased or decreased function as a component of a signal transduction pathway.

Over eighty GEFs and seventy GAPs have been identified, suggesting that Rho GTPase regulation is exquisitely complex. This review will focus on the role of Rho GTPases on neurodevelopement, examine the known GTPase regulators mutated in Intellectual Disability (ID) and discuss emerging opportunities for therapeutic approaches.

## 2. The Molecular and Cellular Processes Controlled by Rho GTPases in the Construction of Neural Network

GTPases of the Rho class are molecular hubs that link extracellular cues with changes in intracellular cytoskeleton dynamics. Changes in cytoskeleton are required to execute cell polarity and cell motility processes such as extending and retracting protrusion, cell migration and change in cell shape [2,3].

Rho GTPases are activated by growth factors, adhesive ligands and guidance cue receptors such as slit, ephrins, netrins, and semaphorins [4,5]. Non-receptor tyrosine kinases, such as Focal Adhesion Kinase (FAK) and Src Family Kinases (SFKs) impact on Rho GTPases and regulate actin dynamic and cell motility [6,7,8,9]. Integrin receptors and adhesion molecules (*N*-cadherin) activate Rho GTPases [10,11]. Activated ion channels, such as α-amino-3-hydroxy-5-methyl-4-isoxazolepropionic receptor (AMPAR) and *N*-methyl-d-aspartate receptor (NMDAR) also impact on Rho GTPases [12].

Cytosolic [Ca^2+^] fluctuations at the growth cone influence Rho GTPase activity and induce changes in the actin cytoskeleton [13,14]. For example, Brain-derived neurotrophic factor (BDNF) and netrin activate [Ca^2+^]-dependent calmodulin kinase II (CaMKII), which increases Rac1/cdc42 and decreases RhoA activity to promote axon outgrowth [15].

Downstream, GTPases regulate several aspects of cytoskeleton assembly/disassembly, such as actin filament polymerization and severing, actomyosin contractility and microtubule elongation [16]. Specifically, RhoA controls nucleation, elongation, branching and severing of the actin filament network via the Rho kinase-LIM domain kinase (ROCK-LIMK) pathway that impacts on the actin-binding protein actin depolymerizing factor (ADF)/cofilin [17,18,19,20]. Rac1 and cdc42 also govern actin cytoskeletal dynamics via ADF/cofilin, however they utilize the p21-activated kinase (PAK)-LIMK pathway [21,22]. Rac1 and cdc42 act on actin dynamic also via WAS protein family member (WAVE) and Wiskott-Aldrich syndrome like (N-WASP), respectively [4]. Actomyosin contractility is regulated by RhoA through the ROCK-myosin light chain (MLC) pathway and by cdc42 via the PAK-MLC kinase (MLCK) pathway [23].

Actin polymerization, increased myosin II motor function and active association of actin-binding proteins with adhesion complexes promote neurite elongation and leading edge progression. The phosphorylation of shootin1 downstream of Rac has been recognized as a key mechanism to couple enhanced actin flow with cell adhesion via a linking bridge, known as a “clutch” [22,24,25].

GTPases also control microtubule elongation [26]: Rac1 and cdc42 use PAK kinases as downstream targets, which can act through Op18 and control microtubule growth. Moreover Rac1 and cdc42 regulate neuronal migration and the formation of leading process of migrating neurons via the activation of the downstream c-Jun N-terminal kinase (JNK)-microtubule pathway through the association with IQ motif containing GTPase activating protein 1 (IQGAP1) [27,28]. Instead RhoA acts on the regulation of microtubule stabilization through Dia [29,30].

The Rho, Rac1 and cdc42 trasduction pathways converge and diverge at different levels of the pathways. All these convergent and divergent pathways are tightly controlled by a complex and multi-layered set of regulators (GAPs, GEFs and other less well understood). For example RhoA, Rac1 and cdc42 promote actin polymerization via LIMK activation, while actomyosin contractility promoted by RhoA is inhibited by Rac1 and cdc42 through inhibition of MLCK [23].

The actions of Rho GTPases depend on the developmental time. At early stages the control of cytoskeletal dynamics is essential for the acquisition of cell polarity, hence for neurogenesis; indeed loss of RhoA results in three distinct cortical malformations: (1) a defect in the proliferation of progenitor cells leading to a bigger cerebral cortex; (2) a change in the morphology of radial glial cells with the formation of a subcortical band eterotopia; (3) an increase in the speed of migrating newborn neurons [31]. At later developmental stages, the control of cytoskeletal dynamics is essential for axonogenesis, dendritogenesis, axon guidance and neuronal migration [3,4], hence required for the construction of the excitatory and inhibitory networks and their complexity. For this reason, changes of the spatiotemporal activity of small GTPases, such as those due to mutations in GAPs and GEFs, affect neuronal migration, dendrite extension and complexity, axon extension and guidance, and spine shape and plasticity, resulting in ID and other cognitive deficits.

Rac and cdc42 are generally associated with promotion of elongation, branching, and complexity, while RhoA is generally associated with the opposite: Inhibition of elongation, branching, and complexity [32]. However this notion is based on in vitro studies that use overexpression of dominant-negative (DN) and constitutively-active (CA) mutants, while in vivo studies have not always confirmed this notion.

In adult neurons, small GTPases control dynamic events of the actin cytoskeleton of the dendritic spine (the postsynaptic compartment) of the excitatory synapse [33,34,35], thus participating in the synaptic plasticity and the maturation of cognitive functions.

Finally, a novel function of small GTPases in the control of ROS production has recently been shown [36,37]. Whether this could represent a unifying mechanisms that participates in the endophenotype leading to ID remains to be further investigated.

### 2.1. GAP and GEF at the Growth Cone

**Figure 1:**
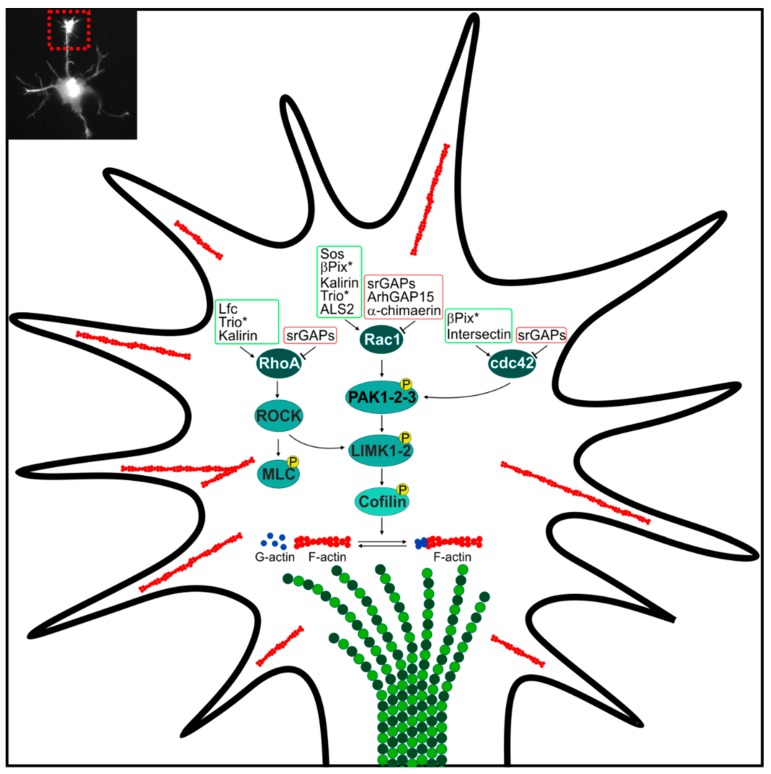
Regulations of Rho GTPases at the growth cone, by GTPase-activating proteins (GAPs) and Guanine nucleotide Exchange Factors (GEFs) implicated in Intellectual Disability. Green and red boxes surround GEF and GAP proteins, respectively. Asterisks indicate that are mutated in Intellectual Disability (ID) and other human diseases comprising ID. Circled P indicates phosphorylation. Arrows indicate activation, T bars indicate inhibition. A representative small magnification image of a growth cone is provided in the inset (top left). ROCK, Rho kinase-LIM domain kinase; MLC, myosin light chain; PAK1-2-3, p21-activated kinase 1-2-3; LIMK1-2, Rho kinase-LIM domain kinase 1-2.

RhoA, Rac1 and cdc42 connect a wide spectrum of external guidance molecules to cytoskeletal changes and thereby regulate the growth cone morphology and dynamic of the growth cone and assure the directed elongation of neuronal processes. A tight regulation through GAPs (such as *α-chimaerin*, *ArhGAP15* and *SrGAPs*) and GEFs (such as *ALS2*, *Sos*, *βPix*, *Kalirin*, *Trio*, *Ephexin1*, *Lfc* and *Intersectin*) is determinant for controlling growth cone protrusion, growth cone collapse and neurite retraction (Figure 1).

α-chimaerin is a GAP for Rac1 that mediates EphrinB3/EphA4 signaling during the formation of motor neuron circuits, via inactivation of Rac1. In cultured hippocampal neurons, EphrinA-induced growth cone collapse is associated with tyrosine phosphorylation of α2-chimaerin and inhibition of the Rac-PAK pathway [38,39].

Trio is essential for netrin1-induced axon elongation and guidance. Trio displays two GEF domains of distinct specificity: Ras guanine nucleotide exchange factor1 (GEFD1) activates the small GTPases RhoG and Rac1, whereas GEFD2 acts on RhoA. Trio-induced neurite outgrowth is mediated by the GEFD1-dependent activation of RhoG, previously shown to be part of the nerve growth factor (NGF) pathway [40]. The chaperone activity of Hsc70 is required for Rac1 activation by Trio and this function underlies netrin-1/DCC-dependent axon outgrowth and guidance [41]. In response to netrin-1 Trio is phosphorylated (Y2622) by Src family kinases, and this step is essential for the regulation of the DCC/Trio signaling complex during axon outgrowth [42].

The chemorepulsive molecule Slit signals via the Robo1/2 receptors. SrGAPs are recruited downstream of this signaling, locally inactivates Rho GTPases thereby reducing actin polymerization asymmetrically, and leading to subsequent turning of the growth cone away from Slit [43]. In response to the Slit/Robo activation, Sos is also recruited to the plasma membrane where it forms a ternary complex with Robo1/2 and the SH3-SH2 adaptor protein DOCK to regulate Rac-dependent cytoskeleton rearrangement [44].

Lfc localizes at the growth cones of developing neurons and negatively regulates neurite sprouting and axon formation via its GEF activity on the Rho signaling pathway. Tctex-1, a dynein light chain implicated in axon outgrowth by modulating actin dynamics and Rac activity, colocalizes and physically interacts with Lfc, thereby inhibiting its GEF activity, decreasing Rho-GTP levels and functionally antagonizing Lfc during neuritogenesis [45].

ArhGAP15 is a Rac1-specific brain-specific GAP and its loss leads to an overall reduced efficiency of neurite elongation and branching, and a simpler morphology of pyramidal and hippocampal neurons [21,25]. Finally, ALS2/Alsin is a Rac1 GEF, that colocalizes with Rac1 within growth cones and promotes neurite outgrowth [46]. Interestingly, ALS2 has been proposed also as a Rac1 effector, thus acting as a bifunctional protein [47].

### 2.2. GAP and GEF at the Leading Edge

**Figure 2:**
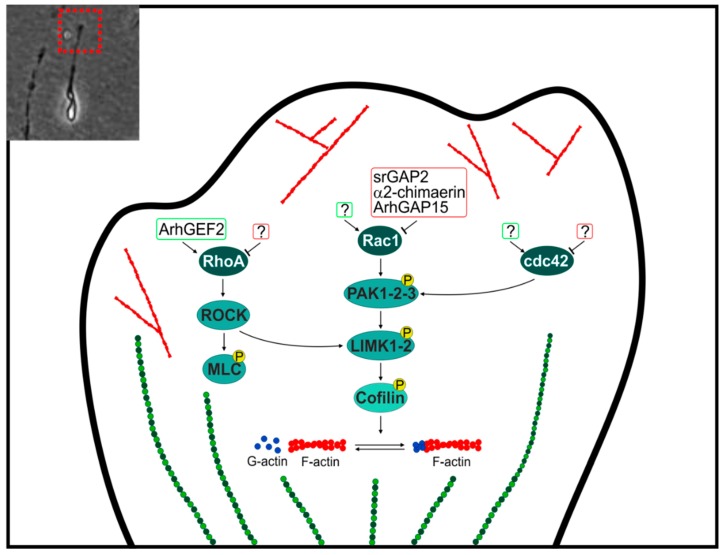
Regulations of Rho GTPases at the leading edge of a migrating neuron, by GAPs and GEFs implicated in Intellectual Disability. Green and red boxes surround GEF and GAP proteins, respectively. Circled P indicates phosphorylation. Arrows indicate activation, T bars indicate inhibition. A representative small magnification image of a migrating neuron with an evident leading edge is provided in the inset (top left). ROCK, Rho kinase-LIM domain kinase; MLC, myosin light chain; PAK1-2-3, p21-activated kinase 1-2-3; LIMK1-2, Rho kinase-LIM domain kinase 1-2.

The molecules that regulate Rho GTPases in this location are illustrated in Figure 2. Rho GTPases present at the leading edge participate in the process of neuronal migration, both radial and tangential. Both types of migration of immature GABAergic neurons are impaired in mice with a combined loss of *Rac1* and *Rac3* [48]. Loss of *ArhGAP15* leads to hyperactive Rac1 pathway and affects the tangential migration of hippocampal interneurons [21,48]. Loss of *α-chimaerin* results in aberrant radial migration and accumulation of ectopic neurons in subcortical regions [49]. *SrGAP2* is required for the efficient production of branches of the leading process [50]. Notably, all these GAP molecules are active on Rac1.

Homozygous frameshift mutations in the *ARHGEF2* gene have been identified as cause of ID. The loss of normal ARHGEF2 activity leads to reduced activation of the RhoA-ROCK-MLC pathway, which in turn is crucial for cell migration. Indeed, *ArhGEF2*^−/−^ mice exhibit altered migration of precerebellar immature neurons [51].

*AUTS2* (Autism susceptibility candidate 2 gene) acts as an upstream factor of Rac1 and cdc42, regulating the cytoskeletal rearrangements in neural cells. Indeed AUTS2 induces lamellipodia in neuroblastoma cells and promotes neurite extensions of cultured hippocampal neurons, via activation of Rac1 [52]. The AUTS2-Rac1 pathway is required for neuronal migration and subsequent neuritogenesis in the cerebral cortex [53]. Conversely, AUTS2 acts as a suppressor of cdc42 and inhibits filopodia formation [53].

The two ROCK isoforms ROCK1 and ROCK2 differentially regulate distinct pathways downstream of RhoA, and their coordinated activities drive cell polarity during migration and synaptogenesis. ROCK1 forms stable actomyosin filament bundles that initiate front-back and dendritic spine polarity. In contrast, ROCK2 regulates contractile forces and Rac1 activity at the leading edge and at the spine head; it also specifically regulates ADF/cofilin-mediated actin remodeling that underlies the maturation of adhesions and the postsynaptic density (PSD) of dendritic spines [54].

RhoG plays a key role in regulating actin dynamics at the leading edge during neural migration, by acting downstream of anillin [55], but its regulation through GAPs and GEFs has been poorly explored.

### 2.3. GAP and GEF at the Dendritic Spine

**Figure 3:**
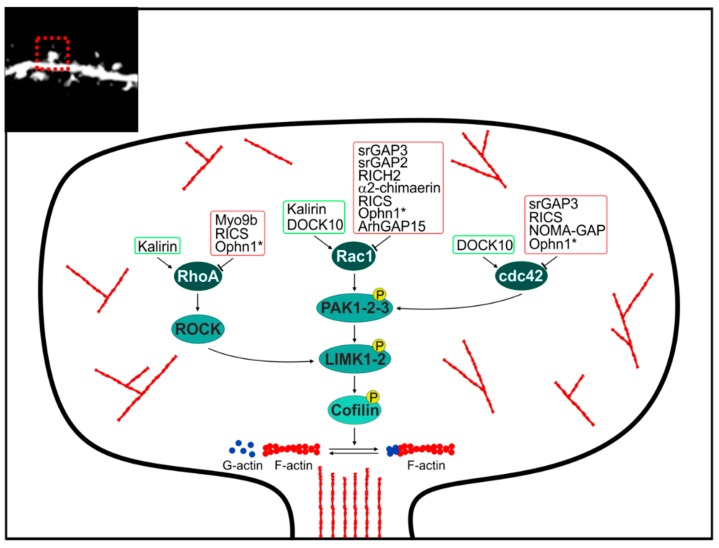
Regulations of Rho GTPases at the dendritic spine of an excitatory synapse, by GAPs and GEFs implicated in Intellectual Disability. Green and red boxes surround GEF and GAP proteins, respectively. Asterisks indicate genes that are mutated in ID and other human diseases comprising ID. Circled P indicates phosphorylation. Arrows indicate activation, T bars indicate inhibition. A representative small magnification image of a dendritic spine is provided in the inset (top left). ROCK, Rho kinase-LIM domain kinase; RICS, Rho GTPase activating protein 32; DOCK10, dedicator of cytokinesis 10; RICH2, Rho GTPase activating protein 44; PAK1-2-3, p21-activated kinase 1-2-3; LIMK1-2, Rho kinase-LIM domain kinase 1-2.

Rho GTPases have been shown to control dendritic spine morphology and plasticity [56]. In general, RhoA activation has a negative effect on spine growth and maturation, whereas Rac1 and cdc42 promote spine formation and maintenance [57]. The specific molecules that regulate Rho GTPases in this location are illustrated in Figure 3. Recent proteomic efforts focused on the PSD place GTPases and their regulatory machinery in a highly connected domain-domain interaction context, linked to several other molecules implicated in neurodevelopmental disorders, including ID [58].

SrGAPs (including SrGAP2 and SrGAP3) are required for the formation of dendritic spines of excitatory synapses, in vivo. Neurons in which SrGAP2 was silenced displayed immature-shaped spines with smaller heads and longer necks, while upon srGAP2 over-expression most spines display an enlarged and mushroom morphology, thus suggesting that srGAP2 is required to promote spine maturation [50]. *SrGAP3* null mice have significantly fewer spines than controls, and even heterozygous mice have fewer mature mushroom-shaped spines [43]. *Wave1* mutant mice, in which the *Wave1* mutation results in loss of binding between Wave1 and SrGAP3, display spine defects [59].

RICH2 is a Rho-GAP which regulates synaptic spine plasticity. RICH2 was identified as an interaction partner of the scaffolding protein SHANK3 at the PSD. In the amygdala of *RICH2* null mice RhoA pathway is hyperactive, actin polymerization is reduced and the density of mature spines is decreased [60]. Their hippocampus and cerebellum display increased multiple spine synapses along with altered receptor composition and actin polymerization [60]. *RICH2* null mice display a significant fear for novel objects and increased stereotypic behavior as well as impairment of motor learning.

Loss of *α2-chimaerin* induces the formation of aberrant polymorphic dendritic spines, acting via Rac1 hyperactivity. Upon loss of α2-chimaerin the complexity of dendritic arbor is reduced and the number of spines that appear poly-innervated is increased [61].

The knockdown of Myo9b or of Rho GTPase activating protein 32 (RICS) results in defects of dendritic morphology, reverted by the inhibition of RhoA/ROCK pathway. These observations provide further supporting evidence for a key physiological function of RhoA in the regulation of dendritic development [62].

Rho GTPase activating protein 33 (NOMA-GAP) acts through the inactivation of cdc42 and its depletion leads to a marked reduction in the number of dendrite branches of layer 2/3 neurons and mislocalization of glutamatergic receptors [63,64,65]. Endocytic recycling of AMPARs at the excitatory synapse is important for the supply of a mobile pool of AMPARs required for synaptic potentiation. This local recycling of AMPARs critically relies on the presence of an endocytic zone (EZ) near the PSD. The precise mechanisms that couple the EZ to the PSD remain still largely elusive, with the large GTPase Dynamin-3 and the multimeric adaptor protein Homer1 as the suspected main players. The PPI between Ophn1 and Homer1b/c is crucial for the positioning of the EZ adjacent to the PSD. Disruption of this interaction causes a displacement of EZs from the PSD, impaired AMPAR recycling and reduced AMPAR accumulation at synapses [66].

Loss of the Rac GEF *Kalirin* results in reduced spine density and reduced dendrite complexity in layer V pyramidal neurons of the frontal cortex [67,68].

Finally, DOCK10 GEF and Ephexin5 are implicated in dendritic spine formation and, the first one acting via Rac1 and cdc42, the second via RhoA [69,70].

The existence of a large number of GAPs and GEFs suggests that some of them are specific to a subcellular compartment and/or they are expressed in different times during development. Only two molecules are shared in all three compartments (the growth cone, the leading edge and the dendritic spine); these are SrGAPs and α-chimaerins [38,39,43,49,61]. This suggests that, while GTPases represent the molecular hubs, the regulation and specificity—hence the adequate cell response—is provided by the regulators. Clearly a better comprehension of the spatiotemporal regulation of Rho GTPases is needed and this could derive from proteomic and interactomic data. Moreover, we note that most studies on Rho GTPases have been done in excitatory synapses, although inhibitory synapses are also plastic. Since excitation/inhibition imbalance is frequently observed in animal models of ID and Autism Spectrum Disorder (ASD), these synapses are evidently important for neurologic and cognitive activities in both the cortex and hippocampus [71,72]. Future studies are needed in this direction.

## 3. Rho GTPases and Intellectual Disability

Intellectual Disability (ID) is a common neurodevelopmental disorder in children, characterized by significant limitations in both intellectual functioning and in adaptive behaviors as expressed in conceptual, social and practical adaptive skills. Estimates of the prevalence of ID among children in the United States based on epidemiologic studies range from 9 to 36 in 1000, depending on the inclusion criteria [73,74,75]. ID is manifested as both syndromic and nonsyndromic forms, depending on whether the disability is associated with other symptoms. A large fraction of ID is linked to the X-chromosome, and are known as X-linked ID, leading to a higher prevalence of ID in males versus females.

Hundreds of mutations have been detected in ID, both syndromic and nonsyndromic. X-linked ID has been associated with mutations in more than eighty genes on the X-chromosome, some of which code for regulators of the small-GTPase family including: *oligophrenin 1* (*OPHN1*), *PAK3*, *Rac/Cdc42 guanine nucleotide exchange factor 6* (*αPIX*), *ARHGEF9*, *FYVE*, *RhoGEF and PH domain containing 1 (FGD1)* and *trio Rho guanine nucleotide exchange factor* (*TRIO*) [71,76,77,78].

Limited evidence is available that the cerebral cortex and the hippocampus of ID children have structural differences. Neuroimaging data suggest differences with limited diagnostic and research value [79,80,81]. Conversely, genetically modified mice are currently the key in vivo approach to investigate the role of gene mutations in ID and related phenotypes, for determining the basic mechanisms, the neurobiological substrates and the neural basis of cognitive function as well as for testing the efficacy of potential therapeutic drugs [82]. Based on a wide spectrum of experimental data from animal models and cultured neurons, it is widely accepted that the cognitive deficits of ID are linked to defects in neuronal networking, synaptic plasticity and the excitation/inhibition balance of the cerebral cortex and hippocampus, and these alterations result in abnormal information processing [83,84,85,86,87,88]. However, a unifying mechanisms is still elusive. In this direction, recent proteomic and interactomic data, obtained from sinaptosomes, intersected with the gene mutations linked to neurodevelopmental disorders including ID, substantially confirm that “GTPase control” is a highly connected molecular hub [58]. 

Here we review in details the functions of the main genes mutated in human ID, analyzing the consequence of these mutations in culture systems and in the mouse models currently available, in terms of neuronal morphology, dendrite and axon complexity, spine shape and density, synaptic physiology and plasticity. Table 1 summarizes the main cellular phenotypes of mouse models of ID, related to altered GTPase functions, focusing on dendrites, axon, spine and synaptic properties.

### 3.1. Mutations of OPHN1

The *Oligophrenin-1* (*OPHN-1*) gene, located on chromosome Xq12, codes for a GAP that negatively modulates RhoA activity by promoting GTP hydrolysis [89]. A number of loss-of-function mutations of the *OPHN1* gene have been detected in patients with mild X-linked ID [90,91,92,93].

Ophn1 is ubiquitously expressed in the developing and adult central nervous system (CNS) [89,90,94,95]. The protein is detected both in glial cells and neurons where it colocalizes with F-actin, notably at the tip of growing dendrites [89] and at both sides of the synapse [66,96,97,98].

In vitro and in vivo studies demonstrated that the loss of *OPHN1* results in increased activity of the RhoA GTPase, and to a lesser extent Rac1 and cdc42, and this affects dendritic tree complexity and synaptic functions of hippocampal neurons [90,98,99,100,101]. Indeed the inactivation of *OPHN1* function induces reduced evoked and spontaneous excitatory postsynaptic currents (EPSCs) and inhibitory postsynaptic currents (IPSCs), associated with decreased readily releasable pool and vesicle recycling, indicating altered neurotransmitter release from the presynaptic processes [94,101].

This altered short-term plasticity is associated with a reduction in mature mushroom-shaped dendritic spines [94,99]. It would be interesting to investigate whether the dysfunction in the neurotransmitter release is the cause or the consequence of the immaturity of dendritic spines in *Ophn1* knock-out (KO) mice.

The loss of *Ophn1* in mice also recapitulates some behavioral, social, and cognitive impairments of the human phenotypes. Indeed *Ophn1* KO mice exhibit behavioral defects in spatial memory together with impairment in social behavior, lateralization, and novelty driven hyperactivity [94].

### 3.2. Mutations of RAC1

*RAC1* is a highly conserved gene, located on chromosome 7p22.1, coding for the small GTPase RAC1 and the neural-specific, developmentally regulated isoform RAC1B [102,103,104]. Rac1 is strongly enriched at the PSD [105].

By exome sequencing of 2104 ID parent/children trios, two non-synonymous *RAC1* mutations were identified which generate dominant-negative (DN) alleles and are expected to result in a condition of haploinsufficiency and hypoactivity of RAC1 [77,78]. Children carrying these mutations also show microcephaly, thus these mutations affect neuronal proliferation. A subsequent study based on whole-exome sequencing (WES) has demonstrated that, depending on where the mutation of *RAC1* occurs, the additional phenotypes observed were: Microcephaly for c53.G > A (pCys18Tyr), c116A > G (pAsn39Ser), c218C > T (p.Pro73Leu) and c470G > A (p.Cys157Tyr) variants, macrocephaly for c151G > A (p.Val51Met) and c151G > C (p.Val51Leu) alleles and normal size for c.190T > G (p.Tyr64Asp) allele [78].

In vitro, the functions of the Rac1 have been studied mostly by expressing DN or constitutively active (CA) mutants, and indicate a critical role of Rac1 in neuritogenesis and neuronal migration [23]. The expression of DN *Rac1* in cultured cortical neurons markedly reduces the number of primary and basal dendrites in neurons with pyramidal morphologies, indicating that Rac is required for the elaboration of dendritic processes [116]. Conversely, the expression of CA Rac leads to the elaboration of dendritic processes [116]. Moreover the expression of DN *Rac1* results in a progressive elimination of dendritic spines, whereas hyperactivation of RhoA causes a drastic simplification of dendritic branch patterns that is dependent on the activity of the RhoA target ROCK [117]. Overall, the results obtained with these in vitro approaches are compromised by the artificial conditions of overexpressed mutant protein and the abundant crosstalk between members of the Rho GTPases family.

In vivo, the full KO of *Rac1* in mice leads to embryonic lethality [118], therefore models of conditional deletion of *Rac1* in the CNS have been generated, including:

*Rac1^flox/flox^; Foxg1-Cre*, leading to an early deletion of Rac1 in the ventricular zone of the forebrain [108];
*Rac1^flox/flox^; Syn1-Cre*, named *Rac1N*, leading to a later deletion of Rac1 in differentiating neurons [111];
*Rac1^flox/flox^; CamKII-Cre*, leading to a brain specific deletion of Rac1 in the hippocampus [110];
*Rac1^flox/flox^; Nkx2.1Tg-Cre*, leading an early deletion of Rac1 in the medial ganglionic eminence (MGE) [119].

The deletion of *Rac1* in ventricular zone (VZ) progenitors does not prevent the axonal outgrowth of telencephalic neurons [108]. However, the anterior commissure is absent, and the corpus callosum as well as hippocampal commissural axons fail to cross the midline in *Rac1^flox/flox^; Foxg1-Cre* KO embryos. The thalamocortical and corticothalamic axons also show defasciculation or projection defects [108]. In contrast to previous studies using DN mutants, these results suggest that Rac1 controls axon guidance rather than neuritogenesis. The specific deletion of Rac1 in the hippocampus induces a reduced spine density [110].

In the hippocampal pyramidal layer of Rac1N mice, Pennucci and colleagues have observed a significant decrease of PV-positive GABAergic presynaptic terminals [111]. They observe no differences in membrane capacitance, input resistance, and membrane resting potential, all of which are passive properties of pyramidal neurons [111]. Instead, hippocampal pyramidal neurons show reduced frequency and amplitude of the sIPSCs [111].

Rac1N mice show generalized hyperactivity and impaired hippocampal-dependent spatial, working and learning and memories [111]. Moreover, quantitative electroencephalography (EEG) analysis revealed impaired synchronization of cortical networks and abnormal brain activity, with slower θ–α rhythms significantly evident in these mice [111].

### 3.3. Rac1 and GABAergic Neurons

Inhibitory GABAergic interneurons play fundamental roles in modulating cortical and hippocampal neuronal circuits [120,121]. The altered neurogenesis and/or migration of these neurons may alter the balance between excitatory and inhibitory activities that is required for proper brain function, a dysfunction thought to be at the basis of various neurological and cognitive conditions including ID, ASD, epilepsy and schizophrenia [122]. 

Several extracellular cues drive the migration and differentiation of the cortical and hippocampal GABAergic cells, while little is known about the intracellular mechanisms that underlie their motility responses [123]. Some recent studies have established that Rac proteins are essentially required for the development of cortical and hippocampal GABAergic interneurons. Rac1 is required for the exit from the cell cycle of the MGE-born interneuron precursors [119] and to confer migratory competence to the differentiating progenitors [108].

*Foxg1-Cre*-mediated *Rac1* deletion severely disrupts the tangential migration of both LGE- and MGE-derived interneurons [108]. Also the *Syn-Cre* mediated deletion of *Rac1* results in defective interneuron migration and differentiation. Interestingly, the hyperactivation of Rac1/Rac3 also affects migration, maturation and synaptogenesis of hippocampal interneurons, in similar ways [21].

Recent works have shown that Rac1 and Rac3 contribute synergistically to the development of cortical and hippocampal GABAergic interneurons [26,48]. These studies have also pointed to a possible role of Rac1 and Rac3 proteins in the later development of specific populations of MGE-derived interneurons [48] and have highlighted cytoskeletal defects in cultured MGE-derived neurons from *Rac1/Rac3* double KO mice that may justify the observed migratory defects [26].

The above observations raise interesting points: The increasing role of inhibitory neurons in neurological and cognitive disorders, and the fact that we know very little about the inhibitory neurons (their subtypes, their connectivity) and in particular their synapses (organization, strength, plasticity). The focus on dendritic spines, i.e., excitatory synapses, justified by the experimental accessibility, leaves several questions unanswered, such as the role of inhibition and the plasticity of non-spiny synapses.

### 3.4. Mutations of PAK3

The p21-activated kinases (PAKs) are a Rac/cdc42-dependent family of Ser/Thr protein kinases. However, PAKs can also act upstream of Rac1 by interacting with the Rac-GEF called PAK-interacting exchange factor (PIX) [124].

In ID patients a missense mutation of *PAK3* gene, located on chromosome Xq23, was found to result in a premature termination of translation, representing a loss-of-function mutation [125,126]. Since these original observations, additional *PAK3* mutations have been detected in both syndromic and non-syndromic ID [127,128,129,130,131], all of which appear to be loss-of-function [132]. 

Pak3 is expressed in the developing and adult brain, including the cortex and hippocampus [125,133,134]. In cultured neurons, active Pak3 is distributed throughout the cell soma and the dendritic shafts [112,133]. In vitro, PAK3 mutations affect actin dynamics at dendritic spines [132] resulting in a decreased density of spines and synapses [135].

Two mouse models have been generated. The first is a DN-*Pak* transgenic mice, consisting in the Pak autoinhibitory domain, which binds to the catalytic domain of group I Paks (Pak1, Pak2 and Pak3) to block their autophosphorylation and consequently the activation of their catalytic activity [112]. The second is the *Pak3* KO [125]. Cortical neurons in these mice display fewer dendritic spines and an increased proportion of larger synapses. Altered synaptic morphology are correlated with enhanced LTP and reduced LTD in the cortex [112]. Notably, Pak3-deficient mice exhibit specific impairments in the consolidation/retention phase of hippocampus-dependent memories [112,133].

### 3.5. Mutations of αPIX

The PAK-interacting exchange factor (αPIX) gene, also known as ARHGEF6 or Cool2, is located on chromosome Xq26.3, codes for a GEF active on Rac1, and can induce membrane ruffling [106,136,137]. In X-linked ID patients the αPIX identified mutation is in the first intron and results in preferential skipping of exon 2 predicting a protein lacking 28 amino acids [138].

Murine *αPix* is highly expressed in the hippocampus, in the cortex and in the cerebellum, both in neuronal cell bodies and in dendrites [106]. In cultures neurons *α*Pix colocalizes with PSD-95 at the PSD of excitatory synapses [136]. The knockdown of *αPix* results in spine morphology alterations, characterized by a decrease of large mushroom-type spines and an increase of elongated spines and filopodia-like protrusions [136,137].

In contrast, the absence of *αPix* in vivo leads to a significant increase in the dendritic length and in the number of dendritic spines along apical dendrites on one side and induces a reduction in excitatory contact density in adult mice on the other side, suggesting that there are more spines that do not participate in the formation of synapses in the hippocampi of *αPix* KO mice [106].

Similar discrepancies between in vivo and in vitro phenotypes have also been noted for Ophn1 and PAK3 [94,99,133,135]. It has been proposed that the cellular environment may account for the distinct phenotypic effects as the environment of neurons and spines is much more complex in vivo compared with dissociated and slice cultures.

Behavioral characterization of *αPix* KO mice revealed largely intact performance in basic tests of spatial reference and working memory. However, these mice exhibit deficits in more complex spatial learning and flexibility, and reduced behavioral control under moderate stress, thus mimicking the human ID phenotype [106].

### 3.6. Mutations of ARHGEF9

The *ARHGEF9* gene, located on chromosome Xq11.1, codes for collybistin, is a neuronal GDP-GTP exchange factor that specifically activates cdc42 and not Rac or RhoA [139]. The first mutation described is a breakpoint between exons 6 and 7 of *ARHGEF9* resulting in the absence of full-length transcripts in patients with ID [140]. Subsequently, Lemke et al. [141] reported a missense mutation in the RhoGEF domain of *ARHGEF9* associated with ID. Using X-chromosome exome sequencing, a novel mutation in *ARHGEF9* was reported in a family with X-linked ID with variable macrocephaly and macro-orchidism [142]. One year later mutations or structural genomic alterations affecting *ARHGEF9* were reported in patients with ID [143].

The mechanism by which mutations of *ARHGEF9* lead to neurodevelopmental disorder is beginning to be clarified; as CB is involved in the formation of inhibitory GABAergic synapses, the loss-of-function mutations of *ARHGEF9* lead to neuronal hyperexcitability. Furthermore, the loss of CB function is associated with reduced inhibition of mTOR [144].

During brain development, alternative *ArhGEF9* mRNA splicing generates CB1 and CB2 isoforms in varying ratios. CB1 level is enhanced during early brain developmental, while CB2 levels remain constant throughout brain development [145].

In primary neurons, CB1 and CB2 differentially promote the formation of gephyrin clusters (hence, GABAergic synapses) depending on the degree of maturity of dendritic segments [145]. During hippocampal adult neurogenesis CB1 regulates neuronal migration, while CB2 is essential for dendrite outgrowth, in fact CB2 overexpression results in a significant reduction in complexity of the dendritic tree and reduced total and terminal dendritic length [145].

ArhGEF9 KO mice show normal locomotor performance but reduced exploratory behavior and enhanced anxiety and impaired spatial learning [115]. These behavioral findings are associated with a region-specific loss of postsynaptic gephyrin and GABA_A_ receptor clusters in the hippocampus and the basolateral amygdala [114,115]. The changes in hippocampal synaptic plasticity are accompanied by increased LTP due to reduced dendritic GABAergic transmission onto CA1 pyramidal neurons [115].

### 3.7. Mutations of FGD1

The gene *FGD1* (*FYVE*, *RhoGEF and PH domain-containing protein 1*), located on chromosome Xp11.22, codes for a protein which binds specifically to the GTPase cdc42 via its PH and DH domains, and stimulates the GDP-GTP exchange of its isoprenylated form [146]. FGD1 is involved in the transmission of signals that regulate the development of axons and dendrites [147]. Mutations in *FGD1* have been associated with a form of syndromic X-linked ID known as the Aarskog syndrome [148] (see below) and with non-specific nonsyndromic X-linked ID [147].

Many missense mutations in *FGD1* in either the structurally conserved region (SCR) or the pleckstrin homology domain (PHD) have been detected in syndromic X-linked ID [149]. Importantly, a base change in exon 4, which results in proline 312 to be substituted with a leucine, is predicted to eliminate a β-turn, creating a long stretch of coiled sequence which may affect the orientations of a SH-3 binding domain and the first structural conserved region [149]. The position of the β-turn is thought to be required for the correct positioning of an AH3 domain 5′ to the relative SCR region 3′. In such way the sequence defined by exon 4 serves as a linker between the FGD1 domain. Although not experimentally verified, such mutations are expected to compromise FGD1 activity.

The microinjection of FGD1 into 3T3 fibroblasts induced actin polymerization and assembly of clustered integrin complexes [150]. Thus FGD1 is involved in the regulation of cdc42 activity at the subcortical actin cytoskeleton and Golgi complex [151].

### 3.8. Mutations of TRIO

The gene *TRIO*, located on chromosome 5p15.2, codes for a large protein of 3097 amino acids, member of the mammalian Dbl family. TRIO comprises two Dbl-homology-Pleckstrin-homology (DH-PH) GEF domains with distinct specificity and a C-terminal serine kinase domains [152]. The first DH-PH domain has been shown to activate Rac1 and RhoG, whereas the second activates RhoA [153,154]. Trio can be alternatively spliced and, as a result, encodes several isoforms whose expression is nervous system specific [155,156].

An intragenic de novo 235 kb deletion of *TRIO* was detected in a boy with ID [113]. Next, targeted sequencing of this gene in over 2300 individuals with ID, identified three additional loss-of-function truncating mutations. The probands featured mild to borderline ID combined with autistic, hyperactive and/or aggressive behavior [113].

Subsequently, a heterozygous frameshift deletion and a de novo missense mutation have been reported in patients with ID associated with microcephaly [157]. The frameshift mutation results in a truncated *Trio* protein that is expected to be degraded by nonsense-mediated decay, thus resulting in the production of a negligible protein product. The truncated TRIO lacks the PH1 domain necessary for efficient GDP/GTP exchange [157].

Trio is highly expressed in the developing and adult brain, including the cerebellum, cortex, hippocampus and thalamus [107,158]. In the rat brain, Trio is expressed during the early postnatal period, but rapidly decreases later on [113]. At the cellular level, ID-associated mutations in TRIO affect dendritic branching and synapse function. Interestingly, upon suppression of endogenous TRIO both synaptic strength and dendritic formation were enhanced [113]. 

In the mouse, the total KO of *Trio* leads to embryonic lethality [159]. Neural-specific deletion of *Trio* leads to reduced extension of granule cell neurites and highly branched processes with perturbed stabilization of actin and microtubules; however *Nestin-Trio* KO mice died before reaching adulthood [107]. In order to address *Trio* gene function in adult mice, *Emx1-Trio*^−/−^ mice have been generated; in this case *Trio* deletion is restricted to the cerebral cortex and hippocampus [158]. These mice show impaired hippocampal-dependent spatial learning ability, while there is no evidence that the memory is affected [158].

### 3.9. Rho-GTPases and Other Neurological/Cognitive Conditions

*RAC* mutations have been detected in children with neurodevelopmental disorders comprising ID [77,78]. Depending on where the mutation on *RAC1* occurs, the ID phenotype was accompanied by microcephaly, macrocephaly or cerebellar abnormalities.

The *βPIX* gene, also known as *ARHGEF7*, located on chromosome 13q34, is ubiquitously expressed in the mouse brain and codes for a cytoplasmic Rac1 GEF protein. By forming a complex with Rac1, βPIX recruits Rac1 to membrane ruffles and to focal adhesions (see Online Mendelian Inheritance in Man (OMIM) *605477). A 1.3 Mb deletion at 13q34, containing *ARHGEF*, was detected by Array Comparative Genomic Hybridization (CGH) in the genome of children with ID [160].

Autism spectrum disorders (ASD) is a group of conditions with a wide range of symptoms and various severity of ID. ASD have been associated with hypoactive *RAC1* function [87,161,162], but also with mutations in the scaffolding molecule genes *SHANK2* and *SHANK3*; the latter is known to modulate the activity of Rac1 and cdc42 via the GEF protein βPIX [60]. In a separate set of ASD patients de novo mutations in *TRIO* have been reported [163]; these mutants tested in rodent neurons turned out to be either the hypo- or hyper-functional variant, and to result in dysregulated glutamatergic synapses. These observations underline how both and excessive or reduced TRIO activity may cause synaptic dysfunction in ASD-related pathogenesis, and point to the TRIO-Rac1 pathway at glutamatergic synapses as a possible key point of convergence of a number of ASD-related genes. In general, these findings support a role of Rho-class GTPases, and RAC1 in particular, in some of the neuropathological events associated with ASD.

Hypoactive Rac has been demonstrated in animal models of depression [164]. Transcriptional profiling of the nucleus accumbens (NAc) for Rho GTPase-related genes, revealed a sustained reduction in Rac1 expression after chronic social defeat stress. This was associated with a repressive chromatin state surrounding the Rac1 promoter. Inhibition of class 1 histone deacetylases (HDACs) rescued both the stress-induced *Rac1* downregulation and the depression-related behaviors.

In a mouse model of Fragile X syndrome (FXS) the Rac1 GTPase pathway was shown to be hyperactive, causing a reduced activity of the actin-depolymerization and severing factor ADF/cofilin which in turn caused spine abnormalities [110,165]. Inhibition of the Rac1 effector PAK1 with a small-molecule inhibitor rescued ADF/cofilin signaling and synaptic signaling in FXS mice [165].

Rho-GTPases have been implicated in Alzheimer’s Disease (AD) [166]. Researchers have analyzed the behavioral modifications in AD mouse models, after modifying Rho-GTPase modulations. It was found that Rac1 activity is increased in AD, while its GTPase-activating protein (GAP) α1-chimaerin, which acts as Rac1 inactivator, is reduced [167]. Rac1 may (con)cause neuropathogenesis of AD, since is regulates the transcription of the *APP* gene (Amyloid β-A4 Precursor Protein, 21q21.3, GRCh38). Notably, in primary hippocampal neurons the Rac1-specific inhibitor NSC23766 was able to decrease both Rac1 activity and APP protein levels in a concentration-dependent manner [168].

Genome analyses on families affected by Aarskog syndrome have revealed a mutation in *FGD1* (R402W at position 1204 (1204C > T) in 20% of the cases [169]. The Aarskog syndrome affects males and is characterized by short stature, craniofacial dysmorphisms, brachydactyly and urogenital abnormalities. The IQ shows a great variability, from normal to severely disabled, and no specific behavioral phenotype has been described so far, even though attention deficit and hyperactivity were observed. Studies performed by Reference [170] reported nine novel mutations (three missense mutations (p.R402Q; p.S558W; p.K748E), four truncating mutations (p.Y530X; p.R656X; c.806delC; c.1620delC), one in-frame deletion (c.2020_2022delGAG) and the first reported splice site mutation (c.1935 + 3A > C)) of *FGD1*, above the 20 distinct abnormalities reported until today. No phenotype-genotype correlations between type and position of mutations and clinical features were noted.

*ARHGAP15* codes for a brain-specific and Rac1-specific GAP, that is able to reduce the GTP-bound level of intracellular Rac1 in the brain. Loss of *ARHGAP15* has been documented in a rare variant of the Mowat-Wilson disease, which is characterized by severe neurological and cognitive deficits, autism and speech impairments [171,172]. The loss of *ArhGAP15* accompanies the loss of the recognized disease gene *Zeb2* [173], nonetheless *ARHGAP15* might contribute to the severity of these conditions or, alternatively, could act as a modifier gene. In mice loss of *ArhGAP15* results in increased Rac1/Rac3 activity, reduced spine density, reduced axonal and dendritic complexity and cognitive deficits [21,25].

The *ARHGAP18* gene, expressed in the developing and adult CNS, controls cell shape and spreading, as well as neuronal motility [174] and has been linked to schizophrenia [175,176]. A genome wide screening strategy was applied along with neuroimaging measures and sixty-one single nucleotide polymorphisms (SNPs) variation was identified in this gene and associated to the phenotypic variation [175].

The gene *SYNGAP1* (*Synaptic RAS-GTPase activating protein1*) located on chromosome 6p.21.32 codes a brain-specific Ras GTPase activating protein localized on dendritic spines in neocortical pyramidal neurons. De novo truncating mutations (K138X, R579X, and L813RfsX22) were detected in children with autosomal dominant nonsyndromic ID [177]. The mutations eliminate the domain for synaptic plasticity and spine morphogenesis and result in the usage of a premature stop codon that destabilizes the *SYNGAP1* mRNA and activates nonsense-mediated decay.

Finally, altered Rac1 signaling has also been implicated in the Rett syndrome, and it may contribute to cyclin-dependent kinase-like5 (CDKL5)-related disorders [178].

### 3.10. Other Mouse Models to Further Explore the ID Cellular Phenotype

LIMKs are a downstream target for RhoA, Rac1 and cdc42, widely expressed in the mammalian CNS [179,180,181]. While LIMK-2 is expressed in all cell types, LIMK-1 is restricted to neuronal tissues and is enriched in mature synapses and in presynaptic terminal of adult neuromuscular junctions and in the spinal cord [182,183,184,185]. The deletion of LIMK-1 in mice lead to a reduction in the level of ADF/cofilin phosphorylation and an increase in its actin depolymerizing activity [109]. These mice exhibit significant abnormalities in both spines and growth cones morphology, in synaptic structure and function, including enhanced hippocampal LTP [109]. LIMK-1^−/−^ mice show altered behavioral responses, including impaired fear conditioning and spatial learning, as indicated by a greater increase in the latency to locate a new platform position during the learning reversal phase [109].

ArhGEF2 (also known as Lfc and GEF-H1) is a Rho GEF protein, and its loss of function is associated to a neurodevelopmental disorder characterized by ID, mild microcephaly and midbrain-hindbrain malformation [51]. In the mouse, ArhGEF2 is highly expressed in cortical and hippocampus neurons, and regulates neurogenesis from cortical precursor cells [186,187]. Cultured hippocampal neurons overexpressing *ArhGEF2* exhibit a greatly reduced dendritic tree with fewer arborizations, decreased spine length and spine area, but increased spine density [187]. The role of ArhGEF2 in vivo has not been reported.

ArhGAP12 is a RhoGAP that negatively regulates Rac1 signaling [188]. *ArhGAP12* is expressed in the hippocampal CA1 region and to a lesser extent in the dentrate gyrus, the protein is detected in the postsynaptic compartment of excitatory synapses of hippocampal CA1 pyramidal neurons [188]. The overexpression of *ArhGAP12* in organotypic hippocampal slices caused a decrease in both spine density and volume, and an increase in immature spines [188], while its silencing resulted in a reduced density of immature spines [188]. At the synaptic level, *ArhGAP12* overexpression significantly depresses AMPAR- and NMDAR-mediated synaptic transmission, while its downregulation resulted in potentiation of AMPAR-mediated but not NMDAR-mediated transmission [188]. In CA1 pyramidal neurons silencing of *ArhGAP12* largely increases both frequency and amplitude of mEPSCs, but had no effect in evoked IPSCs [188]. No differences are observed in presynaptic release [188]. The role of ArhGAP12 in vivo has not been reported.

### 3.11. Specificity of the Rho vs. Rac vs. cdc42 Pathways

Table 2 summarizes the current knowledge on the specificity of the GAPs and GEFs implicated in ID with respect to RhoA, Rac and cdc42. The GAP activity of OPHN1 appears not to be fully specific for RhoA, but to extend to Rac and cdc42 [90]. OPHN1 appears to be mainly active to downmodulate the RhoA/Rho-kinase signaling pathway, repressing its inhibitory activity on endocytosis and actin-myosin contractility [100]. In fact, the inhibition of RhoA and/or ROCK in *Ophn*-mutant mice partially corrects their deficits, thus large part of the ID phenotypes appear to be linked to the hyperactivation of the RhoA/ROCK pathway. The TRIO protein appears to be a GEF for both RhoA, RhoG and Rac1 [153,154], thus its mutations is expected to affect both pathways.

In summary, mutational studies have led to the identification of a number of genes coding for small GTPases of the Rho-class (*RAC1*) or their regulatory proteins (GAPs and GEFs), or their target proteins (PAK, LIMK), whose mutation leads to, or are closely linked to, ID. The currently available knowledge converges to indicate a causative role of hyperactive RhoA pathway and, conversely, hypoactive Rac1 and cdc42 pathways, for the onset of the ID condition.

Mutations directly affecting the small GTPase pathway still account only for a fraction of the total cases of ID, for which the cause remains unknown. Perhaps combinations of disease-associated alleles, each representing only a minor risk factor, will explain some of the remaining (majority) of cases. Whether small GTPases are indirectly involved (i.e., functionally hypoactive for other reasons) in ID in the absence of mutations in known ID genes, is currently unclear.

Altered activity of the Rac1 GTPase is likely to be a common denominator for several neurodevelomental conditions which include an ID component, regardless of whether the genetic cause(s) has been identified. This is not surprising, since these disorders are characterized by altered synaptic plasticity and aberrant spine morphology and density, processes that are regulated by small Rho-GTPases (e.g., [189]). The possibility to finely and specifically remodulate Rac1 activity could have a much wider clinical perspective then those forms of ID caused by Rac1-pathway mutations.

## 4. Cognitive Deficits Due to Developmental Miswiring Can Be Reverted

For a long time, the prevailing view has been that ID and neurodevelopmental disorders in general cannot be cured because the defective cellular processes are difficult to target and to be rescued. Such is perhaps the case of altered neurogenesis and long-distance connections. Much of the treatment has therefore focused on environmental optimization, including individualized education plans, as well as minimizing complicating co-morbidities (such as, visual, sleep or pain co-morbidities) [190]. For specific syndromes associated with ID, therapeutic strategies are known [190]. For instance, for some metabolic disorders, such as Pompe disease, enzyme replacement therapy is used, which can drastically change prognosis and is sometimes accompanied by intellectual sparing.

It is becoming increasingly clear, on the contrary, that errors in local circuitry, neuronal networking and synaptic physiology/plasticity can be partially reverted to a more normal architecture and functioning, accompanied by improvements in cognitive performances, upon correction of the underlying molecular or biochemical defect. This is due to the surprisingly high intrinsic plasticity of short-range projections and synaptic number, position and strength. Although reduced compared to the embryonic and newborn brain, adult local neural circuits are still able to undergo some extent of reorganization and to balance excitatory vs inhibitory activity. Following some notable examples:

### 4.1. Gene Therapy of Rett Syndrome

Rett syndrome is a severe progressive condition comprising ID, due to mutations in the X-linked *MECP2* gene. *Mecp2*B^null/−^ female mice are a widely used model of Rett syndrome [191]. A normal copy of the *Mecp2* cDNA was placed under control of a fragment of its own promoter into scAAV9 vector and tail-vein injected into young adult *MeCP2* null mice. Widespread delivery of MeCP2 was observed, the exogenous MeCP2 protein was found to be functional and able to bind to heterochromatin. Delivery of exogenous MeCP2 restored normal neuronal size and morphology. Strikingly, by 12 weeks and up to 24 weeks, several Rett-associated parameters (including mobility, gait, hindlimb clasping, tremor and general condition) stabilized at an improved level in the scAAV9/MeCP2–injected *MeCP2*B^null/−^ females, whereas control injected females progressed. MeCP2–injected *Mecp2*B^null/−^ mice performed significantly better than controls in the rotarod, inverted grid, and platform tests as well as nesting ability [192]. Thus, gene replacement strategies are effective in reversing the neurological deficits of Rett syndrome.

### 4.2. Channel Therapy for Down Syndrome

The Ts65Dn mice represent the best characterized and mostly used animal model of Down syndrome [193,194]. They recapitulate key hippocampal cognitive deficits of the human syndrome, such as impaired synaptic plasticity, long term potentiation (LTP), learning and memory deficits, and increased generation of forebrain GABAergic interneurons. The latter is believed to lead to imbalanced excitatory/inhibitory transmission in favor of inhibition [195,196], which affects synaptic plasticity and cognition [197,198].

GABA_A_R signaling was found to be excitatory rather than inhibitory in Ts65Dn mice. This excitatory activity was accompanied by (i) a shift in the reversal potential for GABA_A_R-driven Cl^−^ currents (*E*Cl) toward more positive potentials and (ii) increased hippocampal expression of the cation Cl^−^ cotransporter NKCC1 in both Ts65Dn mice and individuals with Down syndrome. The treatment of adult Ts65Dn mice with the FDA-approved NKCC1 inhibitor bumetanide restored *E*Cl to potentials seen in normal mice and rescued both synaptic plasticity and hippocampus-dependent memory [199].

The above observations provide the scientific framework to justify attempts to re-modulate the Rho-GTPases activity and revert the associated neurological and cognitive conditions. Research is increasing in this direction, and it is now clear that re-modulation of the Rho-GTPase pathways impacts on neuronal and synaptic networking also in young adults, both in normal conditions and in pathological contexts. Research on ID, however, is still lagging behind for a number of reasons: *First*, with the exception of the rare inherited forms, the molecular causes are multiple and variable, possibly polygenic and possibly due to complex and unclear gene-environment interactions; *second*, it is often syndromic i.e., associated to other neurological disturbances; *third*, the actual endophenotype associated to nonsyndromic ID in human is unknown, only inferred from mouse models; *fourth*, (as consequence of the three previous ones) we lack suitable cell models, based on human neurons, that reliably recapitulated some of the endophenotypes of ID in the human cortex.

The administration of bacterial Cytotoxic Necrotizing Factor 1 (CNF1), a known GTPase-activating molecule, to normal mice elicited an increased neuronal connectivity via dendritic spine remodeling [33,200]. The same treatment applied to a mouse model of Alzheimer’s Disease (AD) was able to revert some of the behavioral deficits [201]. However, the activity of CNF1 is poorly characterized, and does not discriminate between members of the Rho GTPase family.

The administration of fasudil, an inhibitor of the ROCK and PKA kinase pathways, improved spatial learning and working memory in normal mice and rats [202,203]. Considering that fasudil is safe and well tolerated, and has also been approved for human treatment [204,205,206] to target the CNS [207], it could potentially be used to restore normal cognitive performances in those pathologies linked to RhoA hypoactivity.

### 4.3. Remodulation of RhoA in the Ophn KO Mouse

Hyperactive RhoA pathway due to loss of *OPHN* is the biochemical cause of the complex neuronal phenotypes leading to a XLID (see above). Y-27632 is an inhibitor of the RhoA-dependent kinase ROCK. Treatment of hippocampal slices from *Ophn-1*^−/y^ mutant brains with Y-27632 was able to reverse the synaptic deficits observed in mutant neurons and, on a slower time scale, also the altered dendritic structure [98,101]. A notable feature of this treatment is that it had no effect on WT neurons. The brain penetration of Y-27632 was thought to be too low to achieve therapeutic levels for CNS diseases [208], instead several studies have shown beneficial effects of Y-27632 treatment in animal models of neurodegenerative diseases [209,210,211].

In addition to RhoA/ROCK pathway, in some regions of the *Ophn1*^−/y^ brain increased activity of the Protein Kinase A (PKA) pathway was also detected [98,212]. Fasudil is a compound that is able to inhibit both ROCK and PKA kinases activities. The oral administration of fasudil to adult *Ophn1*^−/y^ mice was able to rescue some of their memory deficits [98]. In a more extensive study, the effect of chronic oral administration of fasudil to adult *Ophn-1*^−/y^ on behavioral and cognitive activities, as well as neuronal and synaptic properties, was examined [213]. The treatment was able to counteract vertical and horizontal hyperactivities, to restore recognition memory, while it had little or no effect on working and spatial memory deficits. The reduced beneficial effect on memory performance suggests that not all neurodevelopmental alterations may be compensated for by treatments at the adult stage. In alternative, it has been proposed that administration of drugs such as antidepressant prior to fasudil could lead to higher efficacy due to their ability to reactivate the juvenile plasticity in the adult brain [214,215]. Globally these results highlight the potential of fasudil treatment in synaptopathies and also the need for multiple therapeutic approaches especially in adult where plasticity is reduced.

Allegra et al. [216] examined the survival, axonogenesis and spinogenesis of hippocampal neurons of Ophn-1^−/y^ mice upon treatment with fasudil. In Ophn-1^−/y^ mice a deficit in neuronal survival has been observed in newborn animals, while proliferation of stem/progenitor cells appears normal. Fasudil treatment was able to increase the number of mature newborn neurons in Ophn-1^−/y^ mice, thus the inhibition of ROCK/PKA may overcome the deficits caused by Ophn1 mutation at an early stage of neuronal maturation. The same authors found altered morphological maturation of newly generated cells with a robust impairment of axonal extension and immature dendritic spines in Ophn-1^−/y^ mice. In this context, fasudil corrected the dendritic spine deficit 21 days after injection, but was unable to restore a normal proportion of newborn neurons projecting the CA3 area. Altogether, the morphological analysis reveals two key processes impacted by Ophn1 deficiency: Axonal extension and dendritic spine morphogenesis, both of which are critical for proper integration of newborn neurons. The fasudil rescue experiments indicate that these two processes proceed via at least partly independent pathways: Dendritic spine density can be restored by downregulating abnormally high ROCK/PKA activity in Ophn-1^−/y^ mice, while the same approach is not effective on aberrant axonogenesis.

### 4.4. Remodulation RhoA in Mouse Model of Rett Syndrome

Positive remodulation of brain Rho GTPases by CNF1 reshapes the actin cytoskeleton and enhances neurotransmission and synaptic plasticity in mouse brains. Indeed a single CNF1 Intracerebroventricular injection (icv) inoculation of CNF1 in a mouse model of Rett syndrome markedly improved the behavioral phenotype of MeCP2-308 mice [217]. CNF1 also dramatically reversed the evident signs of atrophy in astrocytes of mutant mice and restored wt-like levels of this cell population [217]. These results indicate that remodulation of Rho-class GTPases by CNF1 may constitute a totally innovative therapeutic approach for RTT and, possibly, for other disorders associated with ID.

Increasing evidence suggest that mitochondrial dysfunction [218] and deviations from normal Rho GTPases activation state [219] disrupt cognition and synaptic plasticity and may represent important factors in the cellular pathogenesis of ID, including the Rett syndrome. De Filippis et al. [88] achieved a re-activation of Rho GTPases by icv administration of CNF1 to adult *Mecp2-308* heterozygous female mice. They observed a restored mitochondrial ATP production via oxidative phosphorylation, accompanied by the rescue of deficits in spatial reference memory, synaptic plasticity (LTP) and Tyr1472 phosphorylation of GluN2B, which was abnormally enhanced in the hippocampus of Rett model mice [88]. This study provides the first evidence that these brain alterations may be intimately interconnected, thus providing further support to the therapeutic potential of drugs targeting Rho GTPases and their downstream effectors.

### 4.5. Remodulation of Rac1

Rac1 activity is dysregulated in certain neurodevelopmental disorders that present all these three alterations: ID, atypical synaptic plasticity and aberrant spine morphology (see above). Thus, to develop novel therapies for rescuing cognitive impairment, a reasonable approach might be to target Rac1 or its direct regulators.

Modulation of Rac1 activity using chemical inhibitors might be a strategy to reestablish cognitive function. Drugs that regulate Rac1 activation and function could be used to modulate actin cytoskeleton and spine dynamics, representing potential candidates to alleviate the ID condition, whether alone on as part of other disorders associated with spine abnormalities.

Several compounds have been described as Rac1-specific inhibitors [87] including NSC23766, EHop-016, AZA1 and EHT1864. The initial strategy for the identification of these compounds has been primarily based in the information on structure-function of Rac1 interacting with its GEFs [220]. These inhibitors have mainly been tested in models of cancer metastatization, in vitro and in vivo, attempting to inhibit tumor cell migration [87]. Indeed, an elevated level and hyperactivation of Rac1 has been associated with the metastatic potential of tumor cells [221]. 

Little research has been done on the possibility to inhibit the activity of Rac in the CNS. NSC23766 acts as Rac1 inhibitor interfering with specific Rac1-GEFs, Trio and Tiam-1, thus blocking GDP/GTP exchange [222]. Many in vivo and in vitro studies have reported its efficacy in neuronal cells affecting dendritic spine morphology, thus increasing the number of immature spines [223]. Moreover, Zhang et al. [224] have reported a change in neuronal spine density in the rat hippocampus treated with NSC23766. Interestingly, the inhibition of Rac1-associated signaling by NSC23766 has been shown to be involved in extinction of memory [225]. The use of NSC23766 as a therapeutic agent is discouraged due to its high IC50.

EHT1864 selectively inhibits Rac1 downstream signaling by affecting the displacement of GTP. In this manner, Rac1 remains in an inactive state, as Rac1-GDP. In vitro experiments demonstrate that treatment with EHT1864 reduces NMDAR density in rat cortical cultures [226] and decreases density of dendritic spines in coltured hippocampal pyramidal neurons [227]. Further in vivo analysis need to be performed to confirm EHT1864 as a potential therapeutic agent.

The pharmacological regulation of Rac1 in the brain can be effective to restore some of the cellular phenotype underlying ID. However, the available molecules primarily act on the Rac1-downstream pathway without any tissue-specificity and it is overtly clear that the cytoskeleton coordinates a wide number of processes, such as proliferation, morphogenesis and motility, that are finely regulated in every type of cells. Thus, targeting Rac1in this way, in the entire organism to improve cognitive functions may reasonably cause relevant side effects.

As illustrated above, ID and disorders such as ASD which frequently include ID, are consistently characterized by reduced (but not absent) activity of the Rac1 and cdc42 pathways, as opposed to increased RhoA activity (Table 1 and Table 2). This raises the interest to identify positive Rac1 and cdc42 modulators. A re-activation strategy is feasible in those cases in which a quote of Rac1 or cdc42 enzyme is still present in the diseased neurons. Specifically, (a) in the case of heterozygous *RAC1* mutations, the remaining normal quote of endogenous RAC1 can be exploited [78]; (b) in the case of *ARHGEF6* mutations, a normal quote of endogenous RAC1 is still present; (c) mutations in *ARHGEF9, FGD* and *TRIO*, all of which are upstream of Rac1 and cdc42, alter GTPase activity in the presence of normal Rac1 and cdc42. Only the *PAK3* mutations might not to be reverted by Rac1 reactivation.

Interfering with protein:protein interaction (PPI) between GTPase and its GEF is expected to result in their down-modulation [222] while interfering with PPI between GTPase and its GAP is expected to result in their up-modulation [21]. A new generation of Rac1 inhibitors is emerging, identified via structural modelling of the Tiam::Rac1 PPI, which turn out to be effective and selective [228]. In a similar way, PPI between Rac1 and its specific GAPs could be exploited to design peptide or virtual screen for small molecules [229]. Notably, ArhGAP15 is a brain specific, Rac1-specific GAP which modulates by 2-folds neuronal Rac1 activity [21,230]. Clearly, the specific activity and function of each GAP and GEF in brain development is only partly known, and intense research is needed in this direction. 

## 5. Concluding Remarks

Treatments of ID are currently based on environmental optimization and personalized education plans, as well as minimizing complicating co-morbidities. Pharmacotherapy is substantially lacking. Recent evidence suggests that some phenotypes associated with learning disabilities can be reversed, through either genetic correction or pharmacotherapy. At present, whether these provide a realistic opportunity for treatment remains to be proven.

Rho GTPases are a good target for pharmacological intervention, and in specific conditions with genetic mutations causing hyperactive Rho or hypoactive Rac pathways, these would need to be negatively or positively re-modulated, respectively. To achieve brain-specific, GTPase-specific, modest and controlled re-modulation, the PPI between GTPases and their GEFs and GAPs regulatory partners open a promising window of opportunity. However, the role of each of these regulators would need to be defined, in biochemical, cellular and developmental terms. Importantly, novel druggable targets might be identified from studies focusing on the extended protein network linked to Rho GTPases during cell migration and/or neuritogenesis (see [231,232]).

The knowhow needed to identify remodulating compounds based on PPI is here: Structural information on GTPases, GAPs and GEFs, advanced protein-docking tools, effective techniques for peptide design and virtual screens. Thus, taking in account the feature of this type of interaction, PPI-based design of peptides or small molecules seems a most promising strategy, in order: (1) to be tissue-specific; (2) to be GTPase-specific; (3) to achieve mild and finely tuned over- and under-activation.

Translation to the human setting will require the generation and validation of cellular models of ID based on human neurons, that ideally should consent the analysis of both excitatory and inhibitory neurons, and should recapitulate the ID endophenotype. The use of human iPSC seems to be the way to go. Having such a model in hand, this will be used to better define the cellular phenotype of ID in human cells, to identify valuable readouts for a possible correction, and to carry out screening campaigns towards the identification of lead compounds that alleviate ID.

## Figures and Tables

**Table 1:** The main cellular phenotypes of ID mouse models, related to altered GTPase functions, focusing on dendrites, axon, spine and synaptic properties.

Gene Mutated in ID	Genetic Mouse Models	GTPase Pathway Activity ^(1)^	Major Phenotypes ^(2)^	References
**Dendrite and Axon Development**				
**Oligophrenin1**	*Ophn1^−/y^*	↑ RhoA	↓ dendritic tree complexity of dentate gyrus granule neurons	Powell et al., 2012 [101]
**α-PIX (ArhGEF6)**	*α-Pix* KO	↓ Rac1 and cdc42	↑ dendrite length in CA1 hippocampus	Ramakers et al., 2012 [106]
**TRIO**	*Trio^flox/flox^; Nestin-Cre*	↓ Rac1, RhoG and RhoA	Short and highly branched processes of cerebellar granule cells	Peng et al., 2010 [107]
			↓ axon length and irregular growth cone of cerebellar granule cells	
**Rac1**	*Rac1^flox/flox^; Foxg1-CRE*	↓ Rac1	↑ number of primary neurites and secondary branches in hippocampal neurons	Chen et al., 2007 [108]
			Absence of the anterior commissure	
			Corpus callosal axons fail to cross the midline	
			Defasciculation of thalamocortical and corticothalamic axons and projection defects	
**LIMK**	*LIMK-1* KO	↓ Rac1, cdc42 and RhoA	↓ size of the growth cone of hippocampal neurons	Meng et al., 2002 [109]
**Spine Density and Spine Morphology**				
**Oligophrenin1**	*Ophn1^−/y^*	↑ RhoA	↓ density of mushroom-shaped spines on apical dendrites of CA1 pyramidal neurons of the hippocampus	Khelfaoui et al., 2007 [94] Powell et al., 2012 [101]
			↓ length of spines on basal dendrites of CA1 pyramidal neurons of the hippocampus	
			↓ density of mushroom-shaped spines of dentate gyrus granule neurons	
**α-PIX (ArhGEF6)**	*α-Pix* KO	↓ Rac1 and cdc42	↑ spine density in the hippocampus	Ramakers et al., 2012 [106]
**Rac1**	*Rac1^flox/flox^; CamKII-CRE* *Rac1^flox/flox^; Syn1-Cre*	↓ Rac1	↓ spine density in the hippocampus	Bongmba et al., 2011 [110] Pennucci et al., 2016 [111]
			↓ PV-positive GABAergic presynaptic terminals in hippocampal pyramidal layer	
**PAK3**	*dnPAK*	↓ Rac1 and cdc42	↓ spine density of pyramidal cortical neurons	Hayashi et al., 2004 [112]
**LIMK**	*LIMK-1* KO	↓ Rac1, cdc42 and RhoA	Altered spine shape	Meng et al., 2002 [109]
**Synaptic Transmission and Plasticity**				
**Oligophrenin1**	*Ophn1^−/y^*	↑ RhoA	Altered neurotransmitter release in the hippocampus	Khelfaoui et al., 2007 [94] Powell et al., 2012 [101]
			↓ evoked EPSC amplitude and spontaneous EPSC frequency of dentate gyrus granule neurons	
			↓ evoked IPSC amplitude and spontaneous IPSCs frequency in hippocampal slices	
			Impaired vesicle recycling dynamics	
**α-PIX (ArhGEF6)**	*α-Pix* KO	↓ Rac1 and cdc42	↓ synapse density	Ramakers et al., 2012 [106]
			↓ early-phase LTP and ↑ LTD in CA1 hippocampus	
**TRIO**	*Trio* KD neurons	↓ Rac1, RhoG and RhoA	↓ EPSC frequency	Ba et al., 2016 [113]
			↑ AMPAR-mediated synaptic transmission	
			↓ AMPAR endocytosis rate	
**ArhGEF9**	*ArhGEF9* KO	↓ cdc42	↓ postsynaptic gephyrin and GABA_A_ receptor clusters in the hippocampus	Jedlicka et al., 2009 [114] Papadopoulos et al., 2007 [115]
			↓ mIPSC frequency and amplitude of CA1 pyramidal neurons of the hippocampus	
			↑ LTP and ↓ LTD in the hippocampus	
**Rac1**	*Rac1^flox/flox^; Syn1-Cre*	↓ Rac1	↓ frequency and amplitude of the sIPSCs of hippocampal pyramidal neurons	Pennucci et al., 2016 [111]
			Impaired synchronization of cortical networks and abnormal brain activity	
**PAK3**	*dnPAK*	↓ Rac1 and cdc42	Altered presynaptic structure in the cortex	Hayashi et al., 2004 [112]
			↑ AMPAR- and NMDAR-mediated synaptic transmission in the cortex	
			↑ LTP and ↓ LTD in the cortex	
**LIMK**	*LIMK-1* KO	↓ Rac1, cdc42 and RhoA	↑ LTP in the hippocampus	Meng et al., 2002 [109]
			Faster synaptic depression and ↑ frequency of mEPSCs in the hippocampus	

**^(1)^** RhoA/Rac1/cdc42 GTPase pathway activity as a consequence of the mutation of gene related to ID. ↑ and ↓ indicate an increased and a decreased signaling pathway, respectively. **^(2)^** Abbreviations: AMPAR, α-amino-3-hydroxy-5-methyl-4-isoxazolepropionic acid receptor; EPSC, excitatory postsynaptic currents; IPSC, inhibitory postsynaptic currents; LTD, long-term depression; LTP, long-term potentiation; mEPSC, miniature excitatory postsynaptic currents; mIPSC, miniature inhibitory postsynaptic currents; NMDAR, *N*-methyl-d-aspartate receptor; PV, parvalbumin.

**Table 2:** GTPase specificity of genes involved in human ID.

Gene Mutated in ID	Location	Mutation	Functional Effect ^(1)^	GTPase Specificity	Function	References
**Oligophrenin1 (OPHN1)**	Xq12	(X; 12) (q11; q15) translocation 1-bp deletion	LoF	Mainly RhoA	Repression of Rho-kinase pathway Control of endocytosis Control of actin-myosin contractility	Barresi et al. 2014 [100]
**p21 Protein Activated Kinase (PAK3)**	Xq23	Missense (R67C)	LoF	Rac1 and cdc42	Dendrite development Dendritic spine maturation and synaptic plasticity	Ncbi gene ID 5063 RefSeq 2018 Allen et al., 1998 [125] Bienvenu et al., 2000 [126]
**RHO Guanine Nucleotide Exchange Factor 6 (ARHGEF6, αPIX)**	Xq26.3	IVS1-11T→C	Exon 2 skipping (LoF)	Rac1 and cdc42	Induction of membrane ruffling	OMIM #300267 Ramarkers et al., 2012 [106] Kutsche et al., 2000 [138]
**RHO Guanine Nucleotide Exchange Factor 9 (ARHGEF9)**	Xq11.1	Breakpoint betwee nexon 6 and 7 p.R290H missense mutation c1012C > T; p.R338W	Absence of full-lenght transcripts (LoF)	cdc42	Recruitment of gephryn and receptors in GABAergic and glycinergic synapses	Ncbi gene ID 23229 Kalscheuer et al. 2009 [140] Lemke et al., 2012 [141]
**FYVE, RhoGEF and PH Domain-Containing Protein 1 (FGD1)**	Xp11.22	C934T exon 4	Elimination of a β-turn (LoF)	cdc42	Axon and dendrite outgrowth and complexity	Zheng et al. 1996 [146] Martinez-Castellano 2006 [147] Lebel et al., 2002 [149]
**Triple Functional Domain (TRIO)**	5p15.2	De novo 235 kb deletion p.Arg217*, p.Asp1231Valfs*11 p.Trp1376* Frameshift deletion (pGln1489Argfs*11) De novo missense mutation (p.Arg1428Gln, p.Pro1461Thr, p.Asn1080Ile)	LoF	Rac1, RhoG, RhoA	Axon guidance Neurite outgrowth Cerebellum development	Blangy et al. 2000 [154] Jaiswal et al. 2013 [153] Pengelly et al. 2016 [157] Ba et al., 2016 [113]
**Rho Guanine Nucleotide Exchange Factor 7 (ARHGEF7, β-PIX)**	13q34	1.3 Mb deletion at 13q34	LoF	Rac1	Increase of synaptic Rac activity Increase of dendrite protrusions Induction of membrai nruffling	Ncbi gene ID 8874 Orsini et al., 2018 [160]
**Ras-Related C3 Botulinum Toxin Substrate 1 (RAC1)**	7p22.1	c53.G > A (pCys18Tyr) c116A > G (pAsn39Ser)	DN		Modulation of the cytoskeleton Control on cell growth Control on cell-cycle	OMIM #602048 Lelieveld et al., 2017 [77] Rejinders et al., 2017 [78]

**^(1)^** Abbreviations: LoF, Loss of Function; DN, Dominant Negative.

## Abbreviations

ASD	Autism Spectrum Disorder
CA	Constitutively active
DN	Dominant negative
GAP	GTPase Activating Protein
GEF	Guanosine Exchange Factor
GTPase	Guanosine Tri-phosphate Phosphatase
ID	Intellectual Disability
KD	Knockdown
KO	Knockout
PPI	Protein::Protein Interaction
PSD	Postsynaptic Density
XLID	X-linked Intellectual Disability
